# Eating behaviors and weight loss outcomes in a 12-month randomized trial of diet and/or exercise intervention in postmenopausal women

**DOI:** 10.1186/s12966-019-0887-1

**Published:** 2019-11-27

**Authors:** Caitlin Mason, Jean de Dieu Tapsoba, Catherine Duggan, Ching-Yun Wang, Catherine M. Alfano, Anne McTiernan

**Affiliations:** 1 Public Health Sciences Division, Fred Hutchinson Cancer Research Center, M4-B874, PO Box 19024, Seattle, WA 98109 USA; 2 School of Public Health, University of Washington, Seattle, WA 98195 USA; 3 American Cancer Society, Inc., Washington, DC 20004 USA; 4 School of Medicine, University of Washington, Seattle, WA 98195 USA

**Keywords:** Obesity, Disordered eating, Uncontrolled eating, Binge eating, Emotional eating, Restrained eating, Physical activity

## Abstract

**Background:**

Certain eating behaviors are common among women with obesity. Whether these behaviors influence outcomes in weight loss programs, and whether such programs affect eating behaviors, is unclear.

**Methods:**

Our aim was to examine the effect of baseline eating behaviors on intervention adherence and weight among postmenopausal women with overweight or obesity, and to assess intervention effects on eating behaviors.

Four hundred and 39 women (BMI ≥25 kg/m^2^) were randomized to 12 months of: i) dietary weight loss with a 10% weight loss goal (‘diet’; *n* = 118); ii) moderate-to-vigorous intensity aerobic exercise for 225 mins/week (‘exercise’; *n* = 117); iii) combined dietary weight loss and exercise (‘diet + exercise’; *n* = 117); or iv) no-lifestyle change control (*n* = 87). At baseline and 12 months, restrained eating, uncontrolled eating, emotional eating and binge eating were measured by questionnaire; weight and body composition were assessed. The mean change in eating behavior scores and weight between baseline and 12 months in the diet, exercise, and diet + exercise arms were each compared to controls using the generalized estimating equation (GEE) modification of linear regression adjusted for age, baseline BMI, and race/ethnicity.

**Results:**

Baseline restrained eating was positively associated with change in total calories and calories from fat during the dietary intervention but not with other measures of adherence. Higher baseline restrained eating was associated with greater 12-month reductions in weight, waist circumference, body fat and lean mass. Women randomized to dietary intervention had significant reductions in binge eating (− 23.7%, *p* = 0.005 vs. control), uncontrolled eating (− 24.3%, *p* < 0.001 vs. control), and emotional eating (− 31.7%, *p* < 0.001 vs. control) scores, and a significant increase in restrained eating (+ 60.6%, *p* < 0.001 vs. control); women randomized to diet + exercise reported less uncontrolled eating (− 26.0%, *p* < 0.001 vs. control) and emotional eating (− 22.0%, *p* = 0.004 vs. control), and increased restrained eating (+ 41.4%, *p* < 0.001 vs. control). Women randomized to exercise alone had no significant change in eating behavior scores compared to controls.

**Conclusions:**

A dietary weight loss intervention helped women modify eating behaviors. Future research should investigate optimal behavioral weight loss interventions for women with both disordered eating and obesity.

**Trial registration:**

NCT00470119 (https://clinicaltrials.gov). Retrospectively registered May 7, 2007.

## Introduction

Certain eating behaviors including uncontrolled eating, emotional eating and restrained eating- sometimes referred to as cognitive restraint [1] - are common among women with obesity and may affect weight loss outcomes. Uncontrolled eating refers to a tendency to eat more than usual accompanied by a feeling of loss of control, and is a defining characteristic of binge eating disorder. Emotional eating is the tendency to eat in response to stress or negative emotional states. Restrained eating refers to the voluntary control of eating, or to consciously restricting food intake as a means of controlling weight, which can be dually problematic if excessive restriction leads to subsequent overeating [2–4].

Both binge eating disorder and subclinical binge eating behaviors are positively associated with higher mean BMI in the US population [5–7], and are more common among individuals with overweight or obesity [8, 9]. By one estimate, women with obesity are 9.4 times (OR: 9.41; 95% CI: 4.03–21.95) more likely to report binge eating episodes within the past 12 months than women with normal weight [10]. Among 300 sociodemographically diverse primary care patients (18–65 y) with body mass index (BMI) ≥35 kg/m^2^, 50% had high emotional eating scores and 25% had high uncontrolled eating scores, with women more likely to report both emotional eating and uncontrolled eating compared to men [11].

Group-based behavioral weight loss has been shown to reduce binge eating frequency in adults with obesity and binge eating disorder [12], while “spill over” and indirect effects of exercise have been observed on improvements in emotional eating among women with obesity [13, 14]. These changes were, at least in part, attributable to generalization of self-regulation and self-efficacy changes from an exercise context to an eating context [15]. A recent meta-analysis showed no effect of dietary weight loss on psychological stress [16]; however favorable changes in perceived stress, depression and social support have been reported in weight loss and exercise interventions, including among participants of the Nutrition and Exercise in Women (NEW) trial [17] examined in the present study, and could play a mediating role between lifestyle changes and eating behaviors.

Pre-operative restrained eating and disinhibition have been associated with weight loss outcomes after bariatric surgery [18]. In the LookAHEAD trial [19] participants affected by both overweight/obesity and type 2 diabetes, preexisting binge eating was not a contraindication to intensive lifestyle therapy but new or persistent binge eating did attenuate weight loss [20]. The effect of other maladaptive eating behaviors on adherence and outcomes in lifestyle-based weight loss interventions has been mixed [21–25], likely due to considerable heterogeneity in measurement tools, study populations and intervention design. Whether eating behaviors that do not meet formal diagnostic criteria for disordered eating are a barrier to caloric restriction and exercise recommendations frequently prescribed as part of behavioral weight loss programs is an important consideration in designing effective interventions for obesity treatment.

The purposes of this study were to: 1) assess the effects of separate and combined dietary weight loss and exercise interventions on eating behaviors; and 2) examine the effects of baseline eating behaviors on diet and/or exercise intervention adherence and weight loss outcomes among postmenopausal women who participated in a 12- month randomized trial comparing the effects of dietary weight loss and exercise [26].

We hypothesized that: 1) a dietary weight loss intervention, alone or in combination with aerobic exercise would favorably change eating behavior scores compared to controls; 2) higher baseline binge eating, emotional eating, and uncontrolled eating behavior scores would be inversely associated with intervention adherence and associated with less favorable weight loss outcomes; and 3) higher baseline restrained eating scores would be positively associated with intervention adherence and weight loss outcomes.

## Methods

### Design

This study involved secondary post-hoc analysis of data collected during the Nutrition and Exercise in Women (NEW) study - a 12-month randomized controlled trial conducted from 2005 to 2009 at the Fred Hutchinson Cancer Research Center (FHCRC), Seattle, WA (ClinicalTrials.gov #NCT00470119) [26]. Study procedures were reviewed and approved by the FHCRC Institutional Review Board. All participants provided written informed consent.

### Participants

The study design, recruitment, and intervention methods are reported elsewhere [26]. Eligibility criteria included: 50–75 years of age; BMI ≥25.0 kg/m^2^ (if Asian-American ≥23.0 kg/m^2^); < 100 min/week of moderate physical activity; postmenopausal; not taking menopausal hormone therapy for the past 3 months; no history of a diagnosed eating disorder, breast cancer, heart disease, diabetes mellitus, or other serious medical conditions; fasting glucose < 126 mg/dL; non-smoking; ≤2 alcohol drinks/day; able to attend diet/exercise sessions at the intervention site; and a normal exercise tolerance test.

### Randomization and interventions

Eligible women were randomized by a study coordinator to: 1) dietary weight loss (‘diet’; *N* = 118); 2) moderate-to-vigorous intensity aerobic exercise (‘exercise’; *N* = 117); 3) combined diet and exercise (‘diet + exercise’; *N* = 117); or 4) control (no intervention) (*N* = 87). Computerized random assignment was performed using permuted blocks randomization, stratified according to BMI (< or > 30 kg/m^2^) and self-reported race/ethnicity. To achieve a proportionally smaller control arm, the control assignment was randomly eliminated from each block with a probability of approximately 1 in 4. Intervention staff were blinded to whether women were receiving a single or combined intervention (e.g. diet vs diet + exercise).

The dietary weight loss intervention was a modification of the Diabetes Prevention Program [27, 28] and Look AHEAD [19] lifestyle behavior change programs and is described in detail elsewhere [26, 28, 29]. The intervention was delivered by registered dietitians (RD) with training in behavior modification. Program goals were: 1200–2000 kcal/day based on participants’ baseline weight, < 30% calories from fat, and 10% weight loss by 6 months with maintenance thereafter. Individual and group sessions were designed to develop skills for weight loss including goal setting, self-monitoring, coping strategies and problem solving, but were not designed to specifically address disordered eating [29]. Food logs, weekly weigh-ins and session attendance were tracked to measure adherence. Participants who did not meet their weight loss goal by 6 months were encouraged to continue weight loss efforts, and were offered additional sessions; women who reached their goal were allowed to continue losing weight but were monitored to ensure that their BMI did not drop below 18.5 kg/m^2^.

The exercise intervention was delivered by a certified exercise physiologist. Aerobic exercise progressed to 45 min of moderate-to-vigorous intensity exercise on 5 days/week. Because participants were inactive at randomization, the exercise protocol started at 40% of a participants’ maximal heart rate (determined from baseline VO_2_ max exercise testing) for 16 mins/session and gradually increased to 60–75% of maximal heart rate for 45 mins/session by the start of week 8, where it was maintained for the duration of the study. This gradual ramp up helps prevent injury and is standard in exercise trials involving previously inactive participants. The exercise consisted primarily of brisk treadmill walking, stationary bicycling, or other aerobic machine (e.g. rowing machine or elliptical) chosen by the participant. Participants attended 3 supervised sessions/week at the study facility and exercised 2 days/week at home. Weekly activity logs that included exercise mode, duration, peak heart rate, and perceived exertion at each session were reviewed by study staff and used to track intervention adherence.

Women randomized to diet + exercise received separate sessions and were instructed not to discuss diet during supervised exercise. The control arm was instructed not to change their diet or exercise habits for 12 months.

### Measures

All study measures were obtained at baseline and 12 months, and analyzed by trained personnel who were blinded to the participants’ randomization status. Weekly progress evaluations were made via adherence measures (e.g. weekly weigh-ins, diet record reviews, exercise activity logs). However, no additional progress evaluations were made.

Eating behavior scores were derived from self-reported questionnaires completed by participants at baseline and 12 months. Binge eating was assessed using a subset of the five most predictive items [30] from the Binge Eating Scale originally developed by Gormally et al. [31]. Responses to each item were given a score from 0 to 3 and summed for a possible total of 15 with higher scores indicating more severe binge eating behavior.

The revised 18-item Three Factor Eating Questionnaire (TFEQ-R18) developed by Karlsson et al. [1] was used to assess three dimensions of eating behavior: restrained eating (restricting food intake to manage weight; 6 items), uncontrolled eating (losing control over food intake along with subjective feelings of hunger; 9 items) and emotional eating (lacking ability to resist emotional cues; 3 items). The TFEQ-R18 is a reduced version of Stunkard and Messnick’s [32] original Three Factor Eating Questionnaire (TFEQ) and has been validated in populations with and without obesity. Questionnaire scoring was performed as described by de Lauzon et al. [33]. Item scores ranged from 1 to 4 and subscales were summed into a scale of 0–100. Summed raw scores were normalized as 100*(Raw-Min (Raw))/[Max(Raw)-Min(Raw)], where Raw denotes the summed raw score; Min(Raw) and Max(Raw) are the minimum and maximum possible values of the summed raw score, respectively. Higher scores indicate greater restrained eating, uncontrolled eating or emotional eating.

BMI was calculated from measured weight and height. Waist circumference was measured at the minimal waist. Body composition (% body fat, fat mass, lean mass) was measured using a DXA whole-body scanner (GE Lunar, Madison, WI).

Demographic information, medical history, psychosocial factors including stress, anxiety and symptoms of depression that may be associated with maladaptive eating behaviors [34, 35] or lower intervention adherence [36], lifestyle behaviors including smoking status (never, former, current), dietary intake (via a 120-item self-administered food frequency questionnaire) [37], and 7-day average pedometer daily step count (Accusplit, Silicon Valley, CA) were also assessed at baseline and 12 months. Participants regularly taking prescription antidepressant or anxiolytic medications (e.g., selective serotonin reuptake inhibitors, serotonin-norepinephrine reuptake inhibitors, tricyclics, atypical antidepressants) at baseline were classified as antidepressant/anxiolytic users. Depression and anxiety were assessed by the Brief Symptom Inventory-18 [38] and scored according to the scoring manual [39] with higher scores indicating more symptoms of depression and anxiety. Perceived stress was assessed with the Perceived Stress Scale [40]; scores ranged from 0 to 4 with higher scores indicating greater perceived stress. Overall social support was assessed by the short version of the Medical Outcomes Study Social Support Survey [41]. A mean of all item scores was calculated and converted to a score ranging from 0 to 100. Higher social support scores suggest greater perception of social support.

### Statistical analysis

All available data were used without imputation for missing values, except for the eating behavior variables in cases where missing data on items contributing to a summed raw score were imputed by the mean of the other available items used to calculate the summed raw score. The Shapiro-Wilk, Kolmogorov-Smirnov and Anderson-Darling tests for normality indicated that the eating behavior variables were not normally distributed. Descriptive data are presented as means with standard deviations (SD) or frequencies. Differences in baseline eating behavior scores according to demographic characteristics were tested using two-tailed t-tests. Correlates of baseline eating behavior scores were examined using Pearson correlation coefficients.

The mean change in eating behavior scores between baseline and 12 months in the diet, exercise, and diet + exercise arms were each compared with controls using the generalized estimating equation (GEE) modification of linear regression to account for intra-individual correlation over time. Models were adjusted for age, baseline BMI (< 30, ≥30 kg/m^2^), and race/ethnicity (black, white, other). Subsequent models were additionally adjusted for baseline perceived stress, anxiety, depression, and social support scores which were significantly associated with baseline eating behavior scores. The intervention effects were examined based on the assigned treatment at randomization, regardless of adherence or study retention (i.e., intent-to-treat). We used Bonferroni correction (two-sided alpha: 0.05/3 = 0.017) to adjust for multiple comparisons.

We examined the associations between baseline eating behavior scores and adherence to the diet and exercise interventions using linear regression. To maximize group size, intervention adherence was examined in all women receiving the diet intervention (diet and diet + exercise arms) and in all women receiving the exercise intervention (exercise and diet + exercise arms). These analyses were repeated after excluding participants who did not complete the study. Finally, mean 12-month weight changes were compared between women reporting a decrease versus no change or an increase in eating behaviors within each study arm using two-tailed t-tests. All statistical analyses were performed using SAS software version 9.4 (SAS Institute, Cary, NC).

## Results

**Fig. 1:**
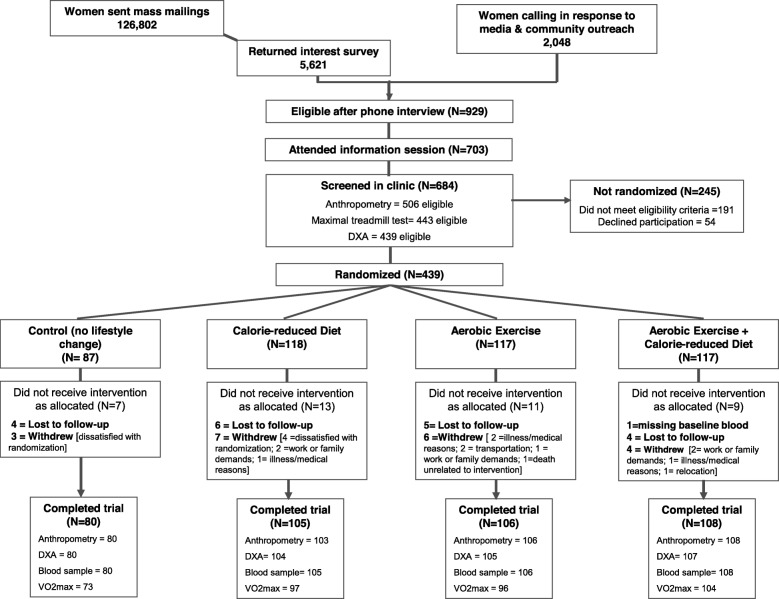
Flow of participants through the Nutrition and Exercise in Women (NEW) trial

At 12 months, 398 of 438 participants completed questionnaires and a physical exam; 39 women did not complete the study (Fig. 1). There were no significant differences in mean baseline eating behavior scores between women who completed 12-month assessments and those who did not (all *p* > 0.30; data not shown).

Mean (SD) baseline scores for specific eating behaviors were: 4.08 (2.86) for binge eating (range: 0–14); 34.84 (17.96) for uncontrolled eating (range: 0–96.30); 48.17 (26.32) for emotional eating (range: 0–100); and 45.20 (15.74) for restrained eating (range: 0–94.44). Differences in baseline eating behavior scores according to demographic characteristics are shown in Table 1. Restrained eating was lower (*p* = 0.04), and emotional eating and binge eating scores were higher (both *p* < 0.001) among women with BMI ≥30 kg/m^2^ versus < 30 kg/m^2^.

**Table 1:** Mean eating behavior scores at baseline according to participant characteristics in the Nutrition and Exercise in Women (NEW) trial

	Binge eating^a^			Uncontrolled eating^b^			Emotional eating^b^			Restrained Eating^b^		
Participant Characteristic	*N*	Mean	SD	*N*	Mean	SD	*N*	Mean	SD	*N*	Mean	SD
Age (years)												
< 60	302	4.12	2.88	303	34.98	17.93	301	48.39	25.23	303	45.29	15.39
≥ 60	131	3.99	2.84	133	34.52	18.09	133	47.66	28.72	133	44.98	16.56
*p*		0.68			0.81			0.80			0.85	
BMI (kg/m2)												
< 30	207	3.49	2.60	208	33.39	17.37	206	42.56	27.01	208	46.81	15.90
≥30	226	4.62	2.99	228	36.16	18.42	228	53.24	24.66	228	43.73	15.48
*p*		< 0.001			0.11			< 0.001			0.04	
Race/Ethnicity												
Non-Hispanic white	369	4.18	2.88	370	35.6	17.87	369	48.93	26.33	370	45.08	15.74
All others	64	3.50	2.70	66	30.56	17.99	65	43.85	26.02	66	45.86	15.82
*p*		0.07			0.04			0.15			0.71	
Smoking status												
Never	255	3.78	2.77	256	33.81	17.42	255	46.80	25.34	256	46.38	15.74
Former	178	4.51	2.94	180	36.30	18.65	179	50.12	27.62	180	43.51	15.62
*p*		0.01			0.16			0.20			0.06	
Current antidepressant/anxiolytic use												
No	286	3.93	2.77	288	33.66	17.58	287	45.63	26.05	288	45.29	15.53
Yes	147	4.37	3.03	148	37.14	18.52	147	53.14	26.22	148	45.01	16.18
*p*		0.14			0.06			< 0.01			0.87	
Completed college degree												
No	149	4.14	2.99	151	33.84	18.57	151	45.14	26.69	151	45.11	16.60
Yes	284	4.05	2.80	285	35.37	17.64	283	49.78	26.02	285	45.24	15.29
*p*		0.75			0.41			0.08			0.94	
Married or living with partner												
No	156	3.96	2.96	158	34.03	17.63	158	48.77	25.43	158	45.8	15.72
Yes	276	4.15	2.81	277	35.33	18.18	275	48.00	26.75	277	44.85	15.79
*p*		0.52			0.46			0.77			0.55	

Differences in mean baseline scores were tested using two-tailed t-tests

^a^Scores derived from a subset of questions from the Binge Eating Scale [30]. Responses to each item of the Binge Eating Scale were given a score from 0 to 3 and summed for a possible total of 15 with higher scores indicating more episodes of binge eating behavior

^b^Scores derived from Three Factor Eating Questionnaire [1]. Subscales of the revised Three Factor Eating Questionnaire (TFEQ-R18) (uncontrolled eating, emotional eating, restrained eating) were summed and normalized into a scale of 0–100. Higher scores reflect more uncontrolled eating, emotional eating, and restrained eating

Baseline correlates of eating behavior scores are shown in Table 2. Higher perceived stress, anxiety, and depression scores were positively associated with binge eating behavior, uncontrolled eating and emotional eating (all *p* < 0.001). Higher social support was significantly inversely associated with these eating behaviors.

**Table 2:** Correlates of eating behavior scores at baseline among participants of the NEW trial

Variable	Binge eating		Uncontrolled eating		Emotional eating		Restrained Eating	
	Rho	*p*	Rho	*p*	Rho	*p*	Rho	*p*
Age	−0.04	0.370	−0.05	0.334	−0.03	0.591	−0.01	0.851
BMI (kg/m^2^)	0.17	< 0.001	0.08	0.107	0.20	<.0001	−0.10	0.031
Waist Circumference	0.15	0.002	0.10	0.033	0.16	0.001	−0.15	0.002
% Body fat	0.12	0.010	0.04	0.366	0.15	0.002	−0.03	0.581
% dietary calories from fat^a^	0.07	0.149	0.10	0.033	0.07	0.125	−0.13	0.007
% dietary calories from carbohydrates^a^	−0.05	0.338	−0.07	0.156	−0.01	0.780	0.01	0.871
average sugar intake (g/day)^a^	0.08	0.100	0.11	0.020	0.14	0.005	−0.08	0.098
average caloric intake kcal/day^a^	0.16	0.001	0.24	<.0001	0.19	<.0001	−0.12	0.011
Perceived stress score^b^	0.29	<.0001	0.23	<.0001	0.31	<.0001	−0.04	0.350
Anxiety score^c^	0.31	<.0001	0.29	<.0001	0.32	<.0001	−0.06	0.217
Depression score^c^	0.31	<.0001	0.24	<.0001	0.33	<.0001	−0.05	0.275
Social support score^d^	−0.14	0.005	−0.16	0.001	−0.16	0.001	0.00	0.951

Associations between baseline characteristics and disordered eating scores were calculated using Pearson correlation coefficients

^a^ Values derived from FFQ were with total daily caloric intake values truncated < 600 kcal and > 4000 kcal

^b^Measured using the Perceived Stress Scale [40]

^c^Measured using the Brief Symptom Inventory [38, 39]

^d^Measured using the short version of the Medical Outcomes Study Social Support Survey [41]

Overall adherence to the dietary weight loss and exercise interventions has been previously described [26]. Using the intent-to treat principle, assuming no weight change among the 40 women without 12-month data, the mean weight change was − 2.4% (*p* = 0.03) in the exercise arm, − 8.5% (*p* < 0.001) in the diet arm, and − 10.8% (*p* < 0.001) in the diet + exercise arm, compared to − 0.8% among controls.

Table 3 shows the 12-month change in eating behavior scores by intervention arm. Compared to controls, women randomized to the diet alone intervention reported significant reductions in binge eating (− 23.7%, *p* = 0.005), uncontrolled eating (− 24.3%, *p* < 0.001), and emotional eating (− 31.7%, *p* < 0.001), and a significant increase in restrained eating (+ 60.6%, *p* < 0.001). Similarly, women randomized to diet + exercise reported significantly less uncontrolled eating (− 26.0%, *p* < 0.001) and emotional eating (− 22.0% *p* = 0.004), and increased restrained eating (+ 41.1%, *p* < 0.001). These results were not meaningfully changed after further adjustment for baseline perceived stress, anxiety, depression, and social support (results not shown). Women randomized to exercise alone had no significant changes in eating behavior scores.

**Table 3:** 12-month change in eating behavior scores* (Mean, SD) according to NEW study intervention arm

	Baseline			12 Months			12-month Change					
	*N*	Mean	STD	*N*	Mean	STD	Change (95% CI)	% Change	P^a^	P^b^	P^c^	P^d^
Binge eating												
Control	87	4.36	3.00	75	4.25	3.30	−0.11 (−0.59, 0.38)	−2.4	*ref*	*ref*		
Exercise	114	3.51	2.84	98	3.49	2.66	−0.02 (−0.46, 0.43)	−0.5	0.939	0.914	0.027	0.029
Diet	117	4.59	2.97	101	3.50	2.56	−1.09 (−1.55, −0.63)	−23.7	**0.005**	**0.005**	0.208	0.177
Diet+Exercise	115	3.91	2.58	104	3.28	2.42	−0.63 (−1.03, − 0.24)	−16.2	0.051	0.054	*ref*	*ref*
Uncontrolled eating												
Control	87	36.68	16.71	76	35.70	17.30	−0.98 (−5.12, 3.15)	− 2.7	*ref*	*ref*		
Exercise	115	31.70	18.05	99	31.07	17.45	−0.63 (−3.22, 1.96)	−2.0	0.985	0.984	**< 0.001**	**< 0.001**
Diet	118	38.56	18.29	101	29.20	16.38	−9.36 (−12.69, −6.03)	−24.3	**< 0.001**	**< 0.001**	0.628	0.576
Diet+Exercise	116	32.79	17.80	104	24.27	14.40	−8.52 (−11.6, −5.44)	−26.0	**< 0.001**	**< 0.001**	*ref*	*ref*
Emotional eating												
Control	87	49.68	26.02	76	48.25	25.37	−1.44 (−6.12, 3.25)	−2.9	*ref*	*ref*		
Exercise	114	44.15	27.87	98	40.48	24.11	−3.68 (−7.79, 0.44)	−8.3	0.378	0.368	0.022	0.024
Diet	118	53.30	25.64	101	36.41	24.55	−16.90 (−21.2, −12.57)	−31.7	**< 0.001**	**< 0.001**	**0.007**	**0.006**
Diet+Exercise	115	45.75	24.99	104	35.68	22.74	−10.10 (−13.0, − 7.14)	−22.0	**0.004**	**0.004**	*ref*	*ref*
Restrained Eating												
Control	87	47.04	14.66	76	46.73	15.76	−0.31 (−3.91, 3.28)	−0.7	*ref*	*ref*		
Exercise	115	47.17	14.50	99	47.85	16.54	0.68 (−2.01, 3.36)	1.4	0.720	0.729	**< 0.001**	**< 0.001**
Diet	118	40.96	15.36	101	65.76	16.65	24.80 (21.04, 28.57)	60.6	**< 0.001**	**< 0.001**	0.065	0.064
Diet+Exercise	116	46.17	17.38	104	65.16	16.23	18.99 (15.31, 22.68)	41.1	**< 0.001**	**< 0.001**	*ref*	*ref*

* Responses to each item of the Binge Eating Scale were given a score from 0 to 3 and summed for a possible total of 15 with higher scores indicating more episodes of binge eating behavior. Subscales of the revised Three Factor Eating Questionnaire (TFEQ-R18) (uncontrolled eating, emotional eating, restrained eating) were summed into a scale of 0–100. Higher scores reflect more uncontrolled eating, emotional eating, and restrained eating

Boldface indicates statistical significance where Bonferroni correction (two-sided alpha: 0.05/3 = 0.017) was applied to adjust for multiple comparisons

p^a^ GEE comparing 12-month change in each study arm compared versus controls

p^b^ GEE comparing 12-month change in each study arm compared versus controls, adjusted for age, race/ethnicity (black, white, other), and baseline BMI

p^c^ GEE comparing 12-month change in diet alone and exercise alone arms versus diet + exercise arm, adjusted for age, race/ethnicity (black, white, other), and baseline BMI

p^d^ GEE comparing 12-month change in diet alone and exercise alone arms versus diet + exercise arm, adjusted for age, race/ethnicity (black, white, other), baseline BMI, perceived stress, anxiety, depression and social support

Overall, baseline eating behavior scores were not statistically significantly associated with diet or exercise intervention adherence, except for higher baseline measures of restrained eating which were associated with the 12-month change in total calories (rho = 0.22, *p* = 0.002) and % calories from fat (rho = 0.17, *p* = 0.01) (results not shown). Select measures of intervention adherence, by study arm, are shown in Table 4. Excluding intervention drop-outs did not meaningfully alter these results.

**Table 4:** Select measures of intervention adherence by tertile of baseline eating behavior scores

	Adherence to diet intervention (mean, SD)			Adherence to exercise intervention (mean, SD)		
	% diet session attended	Δ % calories from fat	Δ total calories	METmins/wk.	Δ mins/wk. MVPA	Δ pedometer step/day
Binge eating^a^						
T1	89.6 (27.1)	−7.5 (6.7)	− 258 (542)	882.9 (431.3)	218 (133)	2935 (2704)
T2	92.1 (25.3)	−7.3 (8.1)	− 336 (494)	921.4 (361.6)	219 (125)	4337 (2872)
T3	92.2 (25.5)	−7.4 (6.9)	− 298 (634)	845.6 (392.5)	211 (117)	3519 (2745)
*ptrend*	0.52	0.92	0.66	0.71	0.81	0.15
Uncontrolled eating^b^						
T1	90.1 (26.3)	−7.6 (6.6)	− 288 (490)	849.6 (363.1)	218 (128)	3656 (2743)
T2	89.0 (26.7)	−7.3 (6.9)	− 226 (527)	945.0 (436.3)	216 (124)	3256 (2763)
T3	95.0 (24.8)	−7.2 (8.5)	− 414 (645)	877.1 (380.6)	219 (128)	4203 (3074)
*ptrend*	0.30	0.74	0.25	0.50	0.97	0.41
Emotional eating^c^						
T1	91.8 (25.0)	−7.4 (6.9)	−346 (526)	904.6 (413.6)	216 (135)	3725 (2595)
T2	89.0 (26.9)	−7.6 (7.7)	− 227 (557)	856.2 (371.7)	217 (123)	3558 (2924)
T3	95.6 (25.4)	−6.6 (6.6)	− 419 (581)	934.8 (413.4)	227 (111)	3492 (3021)
*ptrend*	0.72	0.65	0.93	0.87	0.79	0.68
Restrained Eating^d^						
T1	90.3 (24.4)	−8.2 (6.6)	− 427 (572)	882.9 (431.1)	217 (134)	2935 (2706)
T2	89.1 (29.6)	−7.6 (7.7)	− 182 (558)	921.4 (361.6)	230 (117)	4337 (2872)
T3	94.2 (24.6)	−5.7 (7.8)	−199 (470)	845.6 (392.5)	207 (125)	3519 (2745)
*ptrend*	0.42	0.06	< 0.01	0.71	0.65	0.15

Adherence to diet intervention includes all women randomized to diet and diet + exercise arms; % fat calories were derived from FFQ with total daily caloric intake values truncated < 600 kcal and > 4000 kcal

Adherence to exercise intervention includes all women randomized to exercise and diet + exercise arms

^a^Higher scores reflect more episodes of binge eating. T1: Binge eating score ≤ 2; T2: 2 < Binge eating score ≤ 5; T3: Binge eating score > 5

^b^Higher scores reflect more severe uncontrolled eating. T1: Uncontrolled eating score ≤ 25.93; T2: 25.93 < Uncontrolled eating score ≤ 44.44;

T3: Uncontrolled eating score > 44.44

^c^Higher scores reflect more severe emotional eating. T1: Emotional eating score ≤ 33.33; T2: 33.33 < Emotional eating score ≤ 66.67;

T3: Emotional eating score > 66.67

^d^Higher scores reflect more severe restrained eating. T1: Restrained eating score ≤ 38.89; T2: 38.89 < Restrained eating score ≤ 50; T3: Restrained eating score > 50

Additional exploratory analysis showed that compared to women who reported no change or an increase in maladaptive eating behavior at 12 months, women who reported decreases in binge eating, uncontrolled eating and emotional eating, and increases in restrained eating, generally lost more weight, although not all differences reached statistical significance (Table 5). Significant differences were detected in the diet alone arm between women who experienced an increase or no change in restrained eating compared to those who reported a decrease (− 8.8 kg vs. -3.5 kg, *p* < 0.01), and between those who had a decrease compared to an increase in emotional eating scores (− 9.9 kg vs. -5.1 kg, *p* < 0.001). In the diet + exercise arm, women who reported a decrease in emotional eating lost more weight than those whose scores increased by 12 months (− 10.7 kg vs. -8.3 kg, *p* = 0.03); similarly, women who had a decrease in uncontrolled eating lost significantly more weight than those whose scores increased (− 10.7 kg vs. -7.2 kg, *p* < 0.01).

**Table 5:** Mean (SD) weight loss (kg) according to change in eating behavior scores by study arm

	Control		Diet		Exercise		D + Ex	
	12 month weight change							
	%	Mean (SD)	%	Mean (SD)	%	Mean (SD)	%	Mean (SD)
Binge Eating								
Decrease	39	−3.1 (6.7)	54	−9.3 (6.2)	40	− 1.9 (3.1)	46	−9.9 (5.5)
Increased/No change	61	0.8 (3.0)	46	−7.1 (6.3)	60	−2.5 (4.3)	54	−9.4 (5.8)
*p*		*< 0.01*		*0.08*		*0.47*		*0.66*
Uncontrolled Eating								
Decrease	55	−0.9 (5.1)	62	−9.2 (6.4)	44	−2.7 (4.0)	67	−10.7 (5.9)
Increased/No change	45	−0.5 (5.2)	38	−6.8 (6.0)	56	−2.0 (3.7)	33	−7.2 (4.3)
*p*		*0.70*		*0.06*		*0.42*		*< 0.01*
Emotional Eating								
Decrease	37	−1.8 (5.5)	67	−9.9 (6.6)	40	−2.5 (4.0)	52	−10.7 (6.1)
Increased/No change	63	−0.1 (4.8)	33	−5.1 (4.2)	60	−2.2 (3.8)	48	−8.3 (4.8)
*p*		*0.18*		*< 0.001*		*0.73*		*0.03*
Restrained Eating								
Decrease	46	1.1 (3.1)	9	−3.5 (4.8)	59	−1.63 (3.0)	12	−7.8 (6.1)
Increased/No change	54	−2.3 (5.9)	91	−8.8 (6.3)	41	−2.8 (4.3)	88	−9.8 (5.1)
*p*		*< 0.01*		*0.01*		*0.11*		*0.31*

*p* values were derived from t-tests between women who reported a decrease vs no change/increase in disordered eating scores, by study arm

## Discussion

In our study, women with overweight or obesity who were randomized to a dietary weight loss program that focused on goal setting, self-monitoring, coping strategies and problem solving experienced significant improvements in eating behavior scores compared to controls when the dietary intervention was received alone or in combination with aerobic exercise. Contrary to our hypotheses, baseline eating behavior scores were not strongly associated with intervention adherence or weight loss. However, women who experienced improvements in binge eating, emotional eating and uncontrolled eating behaviors over 12 months had greater mean weight loss than women whose eating behaviors remained the same, or became more severe. As we had hypothesized, restrained eating scores increased significantly among women receiving the dietary intervention and were associated with more weight loss. To our knowledge no participants developed clinically diagnosed anorexia, and none decreased BMI below 18.5 kg/m^2^.

Although our dietary intervention did not specifically address disordered eating per se, these findings are consistent with those that show women with subclinical maladaptive eating behaviors do benefit from standard behavioral weight loss programs [42–44], and confirms the beneficial effect of dietary restraint for weight loss in this sample of women with overweight or obesity. The specific component(s) of the dietary intervention responsible for the observed changes in eating behaviors cannot be known. Baseline perceived stress, anxiety, depression and social support were strong correlates of disordered eating scores at baseline in this study and our results suggest that changes in these variables may be part of the causal pathway. However, these findings need to be confirmed in future studies.

Regular exercise has been shown to reduce symptoms of anxiety and depression [45, 46], but did not significantly change eating behavior scores among women randomized to exercise alone in this trial. Thus, the observed effects may be attributable to changes in these symptoms combined with the dietary counseling on self-monitoring and/or problem-solving received by women randomized to the dietary intervention. Changes in eating behavior scores were not significantly different between diet and diet + exercise arms, except that emotional eating scores were significantly attenuated in women who received both diet and exercise intervention compared to those who received diet alone. The reason for this is uncertain but should be examined in future studies to determine whether this finding has clinical implications.

Previous research has identified maladaptive eating behaviors, especially symptoms of binge eating, as a potential barrier to weight-loss success. After 1 year of intensive lifestyle intervention, individuals aged 45–76 y with type 2 diabetes participating in the Look AHEAD (Action for Health in Diabetes) trial who had stopped binge eating and those who had never reported episodes of binge eating had significantly greater mean weight losses (− 5.3 kg and − 4.8 kg, respectively) than those who continued to binge (− 3.1 kg) or who began binge eating (− 3.0 kg) [44]. In a predominantly male sample of veterans seeking weight loss treatment through the Veterans Health Administration, those who reported no binge eating lost almost twice as much weight and reduced their waist circumference by more than double after 12 months compared to those who reported any binge eating, despite completing treatment at similar rates and attending a similar number of sessions [24]. In other studies, including a combined sample of 44 women who participated in one of three different weight loss studies, binge eating behaviors were a weak prognostic indicator of weight loss success, suggesting that women who binge eat can similarly benefit from standard behavioral weight loss programs as non-binge eaters [42, 43].

Many therapies for binge eating disorders are not designed to assist with weight loss in individuals with comorbid obesity and may not be offered to individuals whose maladaptive eating behaviors fail to reach diagnostic criteria. However, elements of these therapies including cognitive behavioral therapy [47] and mindfulness training [48] can address depression, anxiety and stress, and could be incorporated into behavioral weight loss interventions and tested in future studies.

The current study has several limitations. The TFEQ-R18 was used as a measure of eating behavior because it has been validated in adults with obesity [1]. However, differences in instruments used across studies limits our ability to make comparisons. Additionally, the TFEQ-R18 is a self-reported instrument and therefore participants’ responses may be susceptible to perceptions of social desirability [49] such that behaviors promoted in the intervention might be over-reported, while the inverse would occur for behaviors that were discouraged by the intervention staff or presumed to be negative. Finally, this study population was primarily non-Hispanic white and without a history of a diagnosed eating disorders. Overall, participants reported relatively low (i.e. subthreshold) levels of binge eating and uncontrolled eating and moderate levels of emotional and restrained eating at baseline. Therefore, the present findings may not be widely generalizable beyond this select group of postmenopausal women, including to those with more severe disordered eating symptomology.

Nevertheless, the present study included a large sample over a relatively long 12-month duration with low attrition and was designed to test the separate and combined effects of dietary weight loss and aerobic exercise. This is valuable given that completing moderate amounts of regular exercise can reportedly positively influence mood, self-efficacy, and use of self-regulatory skills (e.g. cognitive restructuring; stimulus control) – three theory-based factors shown to be key predictors of controlled eating and weight-loss success [15, 50]. Yet, we saw no effect of the exercise alone intervention (225 mins/wk. of moderate-to-vigorous activity) on changes in eating behavior scores compared to controls, nor did adding exercise to the diet intervention yield greater changes in eating behavior scores compared to diet intervention alone. Rather, women randomized to diet + exercise reported a smaller change in emotional eating compared to women assigned to diet alone. Thus, efforts to change two behaviors concurrently may limit or alter the types of dietary change participants were able to make. This may be particularly true for women with higher perceived stress, anxiety, and depression scores which were positively associated with binge eating, emotional eating and uncontrolled eating behavior scores at baseline. This is an important area for future study, as is understanding how changes in eating patterns persist over time and influence long-term weight loss maintenance or alter other potentially detrimental compensatory behaviors. Determining whether exercise behavior moderates any such changes is also important to elucidate in order to inform intervention design.

The complex intersection between eating behaviors and obesity represents a challenge for creating effective weight loss interventions. This study shows significant improvements in maladaptive eating behaviors and clinically significant weight loss achieved through participation in a 12-month dietary weight loss program. Yet, behavioral weight loss programs have well documented high rates of attrition and post-intervention weight regain [51]. Whether weight loss outcomes and the long-term success of these programs could be improved by better addressing disordered eating behaviors and their common psychological comorbidities including mood and anxiety disorders, deserves ongoing attention and careful testing in future trials.

## Data Availability

The datasets generated and analyzed during the current study are available from the corresponding author on reasonable request.

## Abbreviations

BMI: Body Mass Index
GEE: Generalized estimating equation
NEW: Nutrition and Exercise in Women
SD: Standard deviation
TFEQ-R18: Revised 18-item Three Factor Eating Questionnaire
